# Antimicrobial stewardship for nurse practitioners and physician assistants: enhancing patient safety through education

**DOI:** 10.1017/ash.2023.434

**Published:** 2023-10-06

**Authors:** Simon Parzen-Johnson, Jacquie Toia, Shan Sun, Sameer J. Patel

**Affiliations:** 1 Departmnt of pediatrics, University of Chicago, Chicago, IL 60637, USA; 2 Division of Infectious Diseases, Ann & Robert H. Lurie Children’s Hospital of Chicago IL 60611, USA; 3 Feinberg School of Medicine, Northwestern University, Chicago, IL 60611, USA

**Keywords:** antimicrobial stewardship, education, infectious diseases, nurse practitioner, pediatrics, physician assistant

## Abstract

**Background:**

As nurse practitioners and physician assistants (APPs) become more prevalent in delivering pediatric care, their involvement in antimicrobial stewardship efforts increases in importance. This project aimed to create and assess the efficacy of a problem-based learning (PBL) approach to teaching APPs antimicrobial stewardship principles.

**Methods:**

A PBL education initiative was developed after communication with local APP leadership and focus group feedback. It was offered to all APPs associated with Lurie Children’s Hospital of Chicago. Participants completed a survey which assessed opinions on antimicrobial stewardship and included knowledge-based questions focused on antimicrobial stewardship. Prescriptions for skin and soft tissue infections associated with APPs were recorded via chart review before and after the education campaign.

**Results:**

Eighty APPs participated in the initial survey and teaching initiative with 44 filling out the 2-week follow-up and 29 filling out the 6-month follow-up. Subjective opinions of antimicrobial stewardship and comfort with basic principles of AS increased from pre-intervention. Correct responses to knowledge-based assessments increased from baseline after 2-week follow-up (p < 0.01) and were maintained at the 6-month follow-up (p = 0.03). Simple skin and soft tissue infection prescriptions for clindamycin went from 44.4% pre-intervention to 26.5% (p = 0.2) post-intervention.

**Conclusions:**

A PBL approach for APP education on antimicrobial stewardship can be effective in increasing knowledge and comfort with principles of antimicrobial stewardship. These changes are maintained in long-term follow-up. Changes in prescribing habits showed a strong trend towards recommended empiric therapy choice. Institutions should develop similar education campaigns for APPs.

## Introduction

Antimicrobial resistance is a public health crisis that will only worsen over time without sustained intervention. Antimicrobial stewardship programs have been shown to optimize antimicrobial prescribing and are recognized as key in preventing the development of resistant organisms.^1^ Education initiatives are regarded as central to successful implementation of stewardship interventions but are considered to be insufficient if implemented alone.^2,3^ However, these initiatives are viewed as an important first step when establishing an antimicrobial stewardship program with a new population.^2^

Nurse practitioners (NPs) have high rates of antibiotic use compared to other prescriber groups, although the rates may differ by subspecialty and practice setting.^4^ However, like physicians, the rate of inappropriate use varies between individual prescribers with substantial areas for improvement.^5–7^ At most institutions, there has been increased emphasis on narrowing the gaps in knowledge about antimicrobial stewardship among MDs and DOs, but NPs are underutilized as collaborators for successful antimicrobial stewardship interventions. Unfortunately, there are limited published data on methodologies and outcomes related to their involvement.^8–13^ Previously published studies have limited their scope to specific practice settings and have small sample sizes.^14^ Given diverse learning backgrounds and differential emphasis on antimicrobial stewardship education in their training, compared to MDs and DOs, it is important to approach education implementation with a collaborative and tailored approach.^15,16^

In this study, we implemented an antibiotic stewardship education curriculum targeted to advanced practice providers (NPs and physician assistants (PAs)). Our course was a combination of lecture followed by problem-based learning (PBL), previously shown to be an effective medical education approach.^17^ We hypothesized that our intervention would improve perceptions of antimicrobial stewardship, as well as improve provider knowledge and antibiotic prescribing for skin and soft tissue infections.

## Methods

### Intervention setting

This study is a prospective study on the implementation and outcomes related to a novel education initiative on antimicrobial stewardship principles directed at NPs and PAs. The study took place within the Ann & Robert H. Lurie Children’s Hospital of Chicago (Lurie Children’s) system between October 1, 2021 and March 1, 2023. Lurie Children’s is a quaternary care children’s hospital, serving the metropolitan Chicago area with a population of approximately 9 million people.^18^ Roughly 80,000 visits are made to the emergency department (ED) and urgent care centers each year. There are more than 600,000 outpatient visits per year to 14 outpatient and surgery centers throughout the region. There is a well-established inpatient antimicrobial stewardship program at the institution, and this program includes regular interval prospective audit and feedback by a dedicated pediatric infectious disease physician and pharmacist.

Within the Lurie Children’s Hospital system, there are over 300 advanced practice providers (APPs).^19^ Upon hire and every 2 years thereafter, APPs and physicians are required to take a brief web-based antimicrobial stewardship training module (duration 5 min) as part of mandatory institutional training. There are several established clinical care guidelines emphasizing antimicrobial stewardship principles that are available to all prescribers in reference to many common pediatric infectious disease diagnoses. In addition, there are many voluntary opportunities for continuing medical education (CME) of APPs at Lurie Children’s including biennial multispecialty APP conferences, financial support for CME activities, professional development courses, and APRN/PA grand rounds. APPs are also invited to, and present at, surgical, medical, and radiology grand rounds.^19^ However, the 5-minute training module is the only required form of antimicrobial stewardship education required at this time.

### Education initiative development and implementation

A team comprised of a pediatric infectious diseases fellow and pediatric infectious disease NP developed the educational component of the initiative. The topics of the lecture were the importance of antimicrobial stewardship, the provider’s role, resources available (ie, antibiograms, antimicrobial susceptibility reports, and antibiotic prescribing recommendations), and how to utilize those resources (Supplement 1). The PBL portion was focused on a case-based implementation of the skills learned with an emphasis on shifting prescription choice for simple skin and soft tissue infections (Supplement 2). A specific emphasis was placed on avoiding inappropriate use of clindamycin given poor local susceptibility rates. The lecture was then presented to the APP leadership group at the hospital for feedback. Once that feedback was incorporated, a focus group comprised of six APPs was created, and the materials were presented for direct participant feedback through unstructured interviews. Advice and recommendations from this focus group were then incorporated into the lecture and PBL component. The initiative was offered on a volunteer basis for all APPs at the institution for continuing education credits. One-hour sessions were held monthly between 4/1/2022 and 9/1/2022.

### Education initiative survey

Prior to completion of the lecture and PBL, all participants were asked to complete a REDCap survey with questions about their area of clinical focus, years of experience, subjective opinions about antimicrobial stewardship, comfort with antimicrobial stewardship principles, and knowledge-based assessments (Supplement 3). A follow-up survey was sent to participants 2 weeks and 6 months after completion of the initial survey. Responses to the surveys were compiled and compared longitudinally.

The survey had 13 questions and took approximately 15 minutes to complete. The first three questions focused on how effectively the institution teaches the importance of antimicrobial stewardship. The following four questions addressed the comfort of practitioners to enact antimicrobial stewardship principles of various types in both the outpatient and inpatient environment. The six knowledge-based questions included two questions about antimicrobial stewardship tools available and four questions focused on appropriate prescribing for skin and soft tissue infections based on local antibiotic susceptibility data.

### Medication prescription assessment

Prescriptions for skin and soft tissue infections associated with APPs, in both the outpatient and inpatient settings, were retrospectively assessed in the 6 months before (10/1/2021–4/1/2022) and after (9/1/2022–3/1/2023) the initiative was implemented. Clinical encounters with ICD-10 codes for cellulitis/acute lymphangitis and cutaneous abscess/furuncle/carbuncle (L03-* and L02-*) were reviewed and included in the study if an APP (1) prescribed the medication and/or (2) wrote the note associated with the clinical encounter. All prescriptions and documentation were individually reviewed and validated by a pediatric infectious disease fellow. Exclusion criteria included prescriptions not associated with an APP, for sepsis, diagnoses other than those of interest, for patients with concurrent antibiotics at presentation for the same diagnoses, significant immunocompromise necessitating alternative antibiotic choice (ie, neutropenic fever and recent solid organ transplantation), or history of known methicillin-resistant *Staphylococcus aureus* (MRSA). The proportion of prescriptions for clindamycin of total prescriptions was calculated in the pre- and post-periods.

### Database operations and approvals

Study data were collected and managed using REDCap electronic data capture tools hosted at Northwestern University.^20,21^ A statement of survey confidentiality was disseminated to all participants, and the option to withdraw at any time was presented. Institutional Review Board and nursing research council approval were obtained for all components of this study.

### Statistical methods

Categorical variables were described with counts and proportions. The primary and secondary outcomes were compared between the two time points using two-proportion z-tests. All statistical analyses were conducted using SAS Enterprise Guide v7.1 (SAS Institute, Inc, Cary, NC).

## Results

### Participant characteristics

Overall, 81 APPs participated in the study. Seventy-one participants were NPs and nine were PAs. The mean experience in their position was 7.0 years, and the median was 4.5 years. The largest divisions represented were cardiology/cardiac surgery, pediatric surgery, critical care, neonatology, oncology, and emergency medicine (Supplement 4). Forty-four participants completed the first follow-up survey and 29 completed the second follow-up survey. Although the surveys were distributed at 2 weeks and 6 months after the initial session, the mean duration of response between the initial assessment and the first follow-up assessment was 21.9 days and between the initial assessment and the second follow-up assessment was 217.4 days. The response rate to the first and second follow-up assessments were 54.3% and 35.8%, respectively.

### Subjective measures

The percentage of participants who reported being extremely or very confident with empiric antibiotic choice and interpreting susceptibility reports increased from the pre-intervention period (15.0% and 22.5%) to the first follow-up (31.8% and 52.3%) and was maintained in the second follow-up period (37.9% and 44.8%) (Table 1). Similar improvement was seen in confidence in both inpatient and outpatient stewardship from the pre-intervention period (15.0% and 15.0%) to the first post-intervention period (36.4% and 27.3%), but there was a decrease seen on the second assessment (24.1% and 13.8%) (Table 1).

**Table 1.** Percentage of participants reporting extremely or very confident in antimicrobial stewardship principles

Category	Pre-intervention (n = 81)	Follow-up 1 (n = 44)	Follow-up 2 (n = 29)	Difference (95% CI)	*P*-value
Empiric choice	15.0%	31.8%		17.0% (1.2%, 32.8%)	0.04
Empiric choice	15.0%		37.9%	23.1% (3.8%, 42.4%)	0.02
Inpatient	15.0%	36.4%		21.5% (5.4%, 37.7%)	<0.01
Inpatient	15.0%		24.1%	9.3% (−8.1%, 26.7%)	0.29
Outpatient	15.0%	27.3%		12.5% (−2.8%, 27.7%)	0.11
Outpatient	15.0%		13.8%	−1.0% (−15.8%, 13.7%)	0.89
Susceptibility	22.5%	52.3%		30.1% (12.7%, 47.4%)	<0.01
Susceptibility	22.5%		44.8%	22.6% (2.4%, 42.8%)	0.03

CI = confidence interval.

When assessing opinions about the effectiveness of our institution’s ability to teach the importance of stewardship in the inpatient and outpatient settings, the percentage of participants who reported extremely or very effective teaching increased with minimal subsequent decreases seen in both groups. (Table 2). Similar increases were seen when assessing how well the institution teaches the importance of patient education in antimicrobial stewardship (Table 2).

**Table 2.** Percentage of participants reporting extremely or very effective institutional teaching of the importance of antimicrobial stewardship by emphasis

Category	Pre-intervention (n = 81)	Follow-up 1 (n = 44)	Follow-up 2 (n = 29)	Difference (95% CI)	*P*-value
Inpatient	33.8%	59.1%		25.8% (8.0%, 43.5%)	<0.01
Inpatient	33.8%		58.6%	25.3% (4.6%, 45.9%)	0.02
Outpatient	21.3%	43.2%		22.2% (5.1%, 39.3%)	0.01
Outpatient	21.3%		41.4%	20.4% (0.4%, 40.4%)	0.05
Patient education	26.3%	52.3%		28.6% (11.1%, 46.2%)	<0.01
Patient education	26.3%		44.8%	18.9% (−1.6%, 39.4%)	0.07

CI = confidence interval.

### Knowledge-based test questions

There was improvement in the percentage of correct answers in the knowledge assessments from baseline (63.5%) to both the first (81.8%) and second (71.8%) follow-up time points (Table 3).

**Table 3.** Percentage of correct responses to knowledge-based assessments

Pre-intervention (n = 486)	Follow-up 1 (n = 264)	Follow-up 2 (n = 174)	Difference (95% CI)	*P*-value
305 (63.5%)	216 (81.8%)		19.1% (12.7%, 25.4%)	<0.01
305 (63.5%)		125 (71.8%)	9.1% (1.1%, 17.0%)	0.03

CI = confidence interval.

### Prescription changes

Clindamycin prescriptions, as a percentage of all prescriptions for simple skin and soft tissue infections, were 44.4% prior to the intervention and 26.5% after the intervention (Table 4).

**Table 4.** Percentage of prescriptions for simple skin and soft tissue infections by antibiotic agent

Agent	Pre-intervention (n = 18)	Post-intervention (n = 34)	Difference (95% CI)	*P*-value
Clindamycin	8 (44.4%)	9 (26.5%)	−18.0% (−45.3%, 9.4%)	0.2

CI = confidence interval.

## Discussion

This study demonstrates a potential strategy for the implementation and assessment of an antimicrobial stewardship curriculum for APPs. There were statistically significant improvements in six out of seven subjective measures relating to antimicrobial stewardship, with only outpatient antimicrobial stewardship comfort not statistically significantly changing after implementation. Knowledge-based assessments statistically significantly improved from pre-intervention to the first post-intervention period with maintenance of improvement in the second post-intervention period. There was also a trend towards improved prescribing habits focused on reduction of clindamycin use in uncomplicated skin and soft tissue infections.

The role of NP has shifted significantly since its inception in 1965.^22^ They are now recognized as key partners in the pediatric healthcare team with contribution to patient education, patient care, and academic research endeavors.^23^ Additionally, there has been continued expansion in the number of states that license NPs to practice independently, without physician association.^24^ Similar practice expansion has occurred with PAs, although it is important to note that their education and roles are significantly different.^25^ Given their increasing impact in healthcare, APPs (NPs and PAs) will need to take on additional responsibility regarding antimicrobial stewardship.

The Center for Disease Control and Prevention and other regulatory bodies have emphasized the importance of nurses and APPs in antimicrobial stewardship, but there have been few publications addressing implementation and outcomes of efforts targeting APPs.^26^ PBL offers a potential methodology that is both engaging and has been shown to improve problem-solving and self-learning skills in medical education.^17^ These skills are key to improving long-term adherence to antimicrobial stewardship teachings. This highlights a major strength of our study in including both short- and long-term follow-up assessments demonstrating maintenance of attitude and knowledge changes. Some of the concerns regarding widespread implementation of PBL-based education models are the increased human resource requirements and the relative inexperience of faculty with guided self-learning.^17^ However, our intervention was effective with only 1 hour of real-time interaction and was both facilitated and implemented by a pediatric infectious disease fellow, thus allaying some of these concerns. Given the research supporting the efficacy of the PBL approach, our findings suggest it can be used in a time- and cost-effective manner to improve antimicrobial stewardship education for APPs at any institution.

It is important to emphasize that education for APPs should be customized to their unique care team roles and previous training. The American Academy of Nurse Practitioners (AANP) highlights that NPs must receive education that is congruent to their population focus and that the education should not be limited to their graduate education programs.^27^ There is a need for postgraduate education in antimicrobial stewardship given that most NP programs do not require additional coursework in microbiology and instead emphasize experiential learning.^15^ There has been a push to include more formal coursework addressing antimicrobial stewardship in university NP programs, but these developments are slow to evolve and would not reach NPs who are more senior and no longer in university courses.^28^ Given these factors, we felt that the most effective model was a collaborative education curriculum with APP leadership, individual clinical APPs, infectious disease NPs, and infectious disease physicians.

The most effective implementation of antimicrobial stewardship requires all prescribers to be incorporated and promote judicious antibiotic use.^26^ Although there has been a call to action within the NP community to advance stewardship knowledge, there has been too little collaboration between physicians and APPs.^19^ An example of such collaborations are APP-run antimicrobial stewardship teams, which have been shown to be effective in antibiotic reduction and narrowing therapy.^29^ Although formal incorporation of APPs into stewardship teams is important, it is also vital to target APPs outside of the field of infectious diseases, such as in our study. To reach a broader audience of APPs, including both outpatient and inpatients providers, infectious disease physicians and pharmacists should partner with APP leadership at an institution to develop directed education and feedback.

APPs bring a unique skillset to the world of antimicrobial stewardship. NP emphasis on holistic care, and experience with bedside care, provides an important perspective to cross-discipline discussions regarding long-term stewardship goals.^27^ APPs are also positioned to alter prescribing habits within a variety of practice settings and subspecialties. They improve patient safety and decrease length of admission within surgical care, two important components of AS.^30^ Additionally, one noted benefit of APPs over residents is the continuity of care they offer by being daily members of the team for longer periods, which again positions them to be effective long-term collaborators in antimicrobial stewardship initiatives.^31^ All of these characteristics demonstrate APPs to be potentially vital members of the antimicrobial stewardship team, and the first step is building this partnership. One of the major successes of our project was seamlessly working with APP leadership to both design and deliver the education initiative, avoiding a paternalistic approach.

One limitation of this study is that education alone usually does not bring about long-term sustained change in altering prescribing behaviors.^3^ However, when combined with long-term sustainable efforts such as prospective audit and feedback, already present at our institution, it can be an effective strategy.^3^ In addition, our stewardship group has developed guidelines that are available to both the inpatient and outpatient providers within the Lurie Children’s Hospital System with updated recommendations on empiric antibiotic choice for skin and soft tissue infections. These guidelines were highlighted as part of the education in this project and could ameliorate some of the concerns about the longevity of change that can be achieved with an educational campaign. Furthermore, although there are more than 300 APPs present at our institution, only 80 APPs took part in the education initiative. Though this is a large sample size for a study of this type, the included APPs did not encompass a large percentage of practitioners at our institution. When assessing prescribing habits, all APPs were included to estimate the effect of the initiative more accurately on overall hospital prescribing habits among APPs, instead of just on the habits of those who took part. This may have contributed to the lack of statistical change between the pre- and post-intervention period and thus may underestimate the effect of the education initiative on individual prescribers. In future endeavors, increasing the participation rate of providers may have a larger impact on prescribing habits. Another limitation of this study is the lack of a comparator group. Given the observational design of the study, it is possible that other hospital initiatives to improve prescribing could have contributed to the changes documented. However, there were no simultaneous initiatives directed at APP attitudes and knowledge about stewardship principles during the study period, decreasing the likelihood of this as a potential confounder.

## Conclusion

Although providers recognize the importance of antimicrobial stewardship, these attitudes are often incongruent with the realities of prescribing practices.^32^ Increasing the appreciation for the immediacy and importance of antimicrobial stewardship efforts can help to improve uptake of interventions that have been shown to be effective in initiating change. Given the trend toward increased prescribing responsibilities for APPs, there must be increased focus on their incorporation into antimicrobial stewardship efforts. Their different education backgrounds necessitate directed and personalized education strategies, while their cross-disciplinary involvement could bring significant new sources of strength to the field. Our study demonstrates that an education initiative, with limited physician time commitment, can make significant headway into the development of both practice changes and individual appreciation of the importance of antimicrobial stewardship. Implementation at an institution level is important, but future initiatives should strive for national-level partnerships and formal integration of antimicrobial stewardship education into APP curricula. As the landscape of pediatric medicine changes to incorporate more APP care, we as a community of practitioners need to continue to adjust and embrace teamwork for the betterment of our patients by including cross-disciplinary education as a major goal for the future.

## Supporting information

Parzen-Johnson et al. supplementary material 1 Parzen-Johnson et al. supplementary material

Parzen-Johnson et al. supplementary material 2 Parzen-Johnson et al. supplementary material

Parzen-Johnson et al. supplementary material 3 Parzen-Johnson et al. supplementary material

Parzen-Johnson et al. supplementary material 4 Parzen-Johnson et al. supplementary material

## Data Availability

All deidentified data are available for review on request from the authors of this publication. This study implements and assesses a PBL approach to education of NPs and PAs in antimicrobial stewardship principles. The role of APPs in pediatric care delivery has increased over the past few decades. Their role in antimicrobial stewardship has often been neglected and is likely an important part of improving antimicrobial prescribing. This study demonstrates the successful implementation of an education initiative at a large tertiary care institution directed at NPs and PAs. The initiative demonstrated improvement in both improved understanding and comfort with antimicrobial stewardship principles.

## Supplementary material

The supplementary material for this article can be found at https://doi.org/10.1017/ash.2023.434
