# Supplementary material for: Antimicrobial stewardship for nurse practitioners and physician assistants: enhancing patient safety through education
Source: Antimicrob Steward Healthc Epidemiol. 2023 Oct 6;3(1):e165. doi: 10.1017/ash.2023.434 (PMC10644159; doi:10.1017/ash.2023.434)
Supplement: Parzen-Johnson et al. supplementary material 1 — Parzen-Johnson et al. supplementary material [file S2732494X23004345sup001.pdf]

Ann & Robert H. Lurie Children's Hospital of Chicago
Northwestern Medicine
Feinberg School of Medicine

APP Antibiotic Stewardship Series: Skin and Soft Tissue Infections

Ann & Robert H. Lurie
Children's Hospital of Chicago
Pediatric Infectious Diseases
April 21st, 2022

BEST CHILDREN'S HOSPITALS U.S.News & WORLD REPORT RANKED IN 10 SPECIALTIES 2021-22

MAGNET RECOGNIZED AMERICAN NURSES CREDENTIALING CENTER

@LuriePedsID
#IDTwitter

Project Origins

- Antimicrobial resistance is a significant problem facing the medical community and world
- Most services in the hospital encounter common infectious disease problems
- Most services at LCH engage APP's in a large proportion of all patient care
- ID education is directed mostly at MD/DO trainees and providers

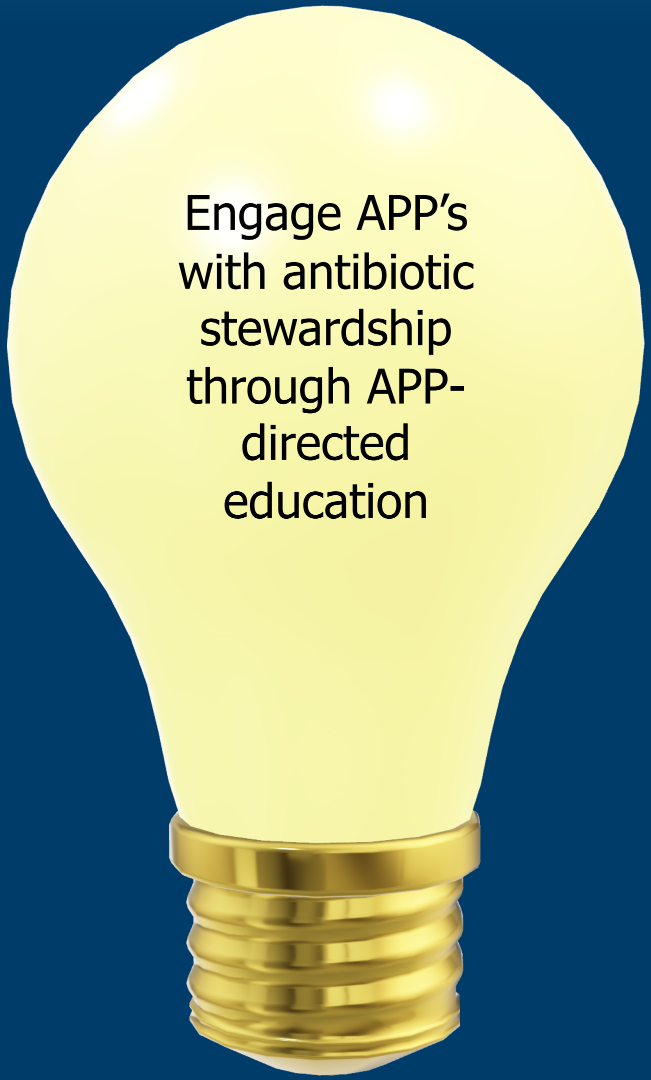

Learning Objectives

- Identify the two most common bacteria that cause skin and soft tissue infections
- Identify the most appropriate first line antibiotic for simple skin and soft tissue infections
- Recognize the utility of institutional and global antibiograms in choosing empiric antibiotics
- Demonstrate how to use microbiology reports to alter therapy based on antibiotic susceptibility

Case Presentation

A 13yo boy comes to clinic because of a rash on his R arm. He thought it was a bug bite, but it continued to grow. He endorses pain, mild swelling and it feels warm to the touch. He denies fever, chills, purulent drainage.

Physical exam is significant for a 4cm area of erythema, without clear borders, that is warm to the touch with mild tenderness to palpation. There is a small area of induration with fluctuance.

You make a diagnosis of cellulitis with abscess.

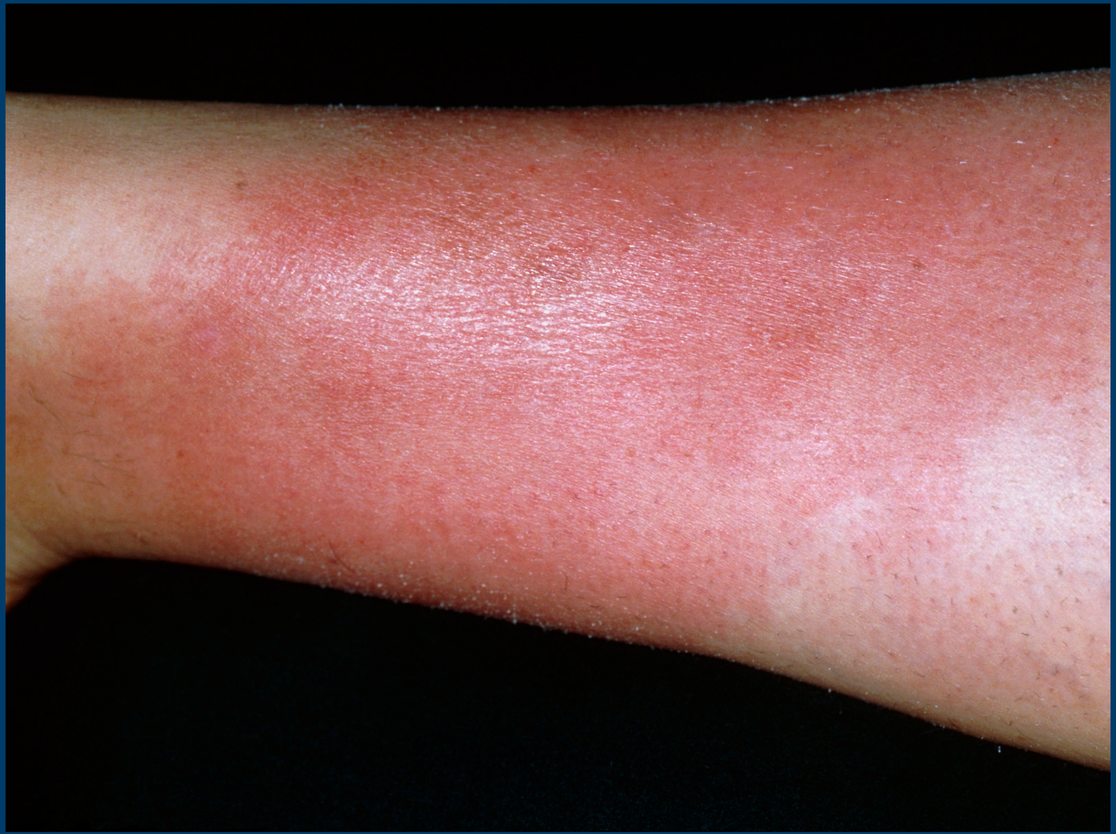

Skin Infections

- Cellulitis = bacterial infection of the hypodermis
  - Fades off into distance
  - *Staph aureus* > *Group A Strep*

- Erysipelas = bacterial infection of the dermis
  - Discrete borders
  - *Group A Strep* > *Staph aureus*

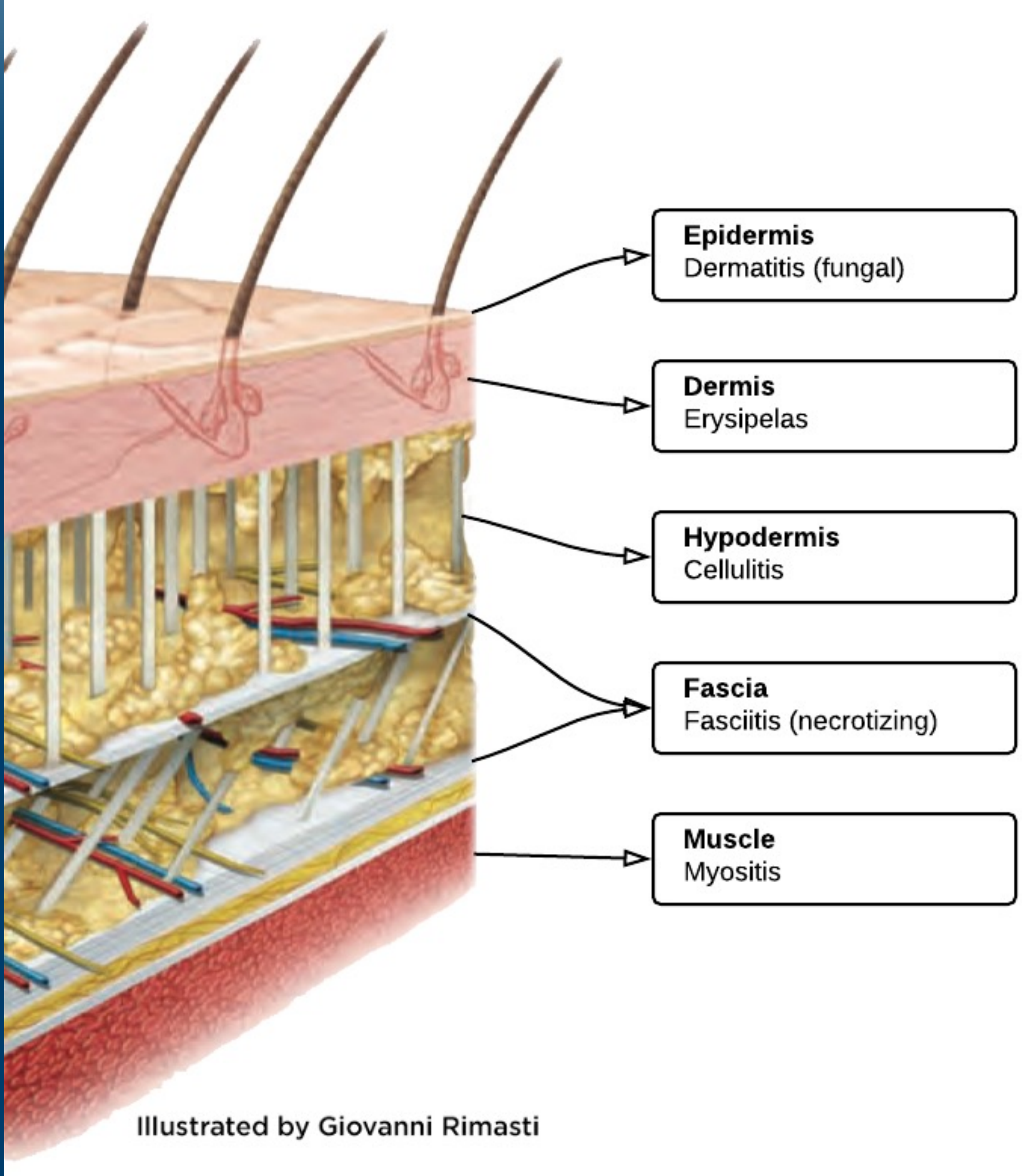

Illustrated by Giovanni Rimasti

Telephone Call | Orders Only | Calculator | Weblinks | Unit Census | ED Track Board | Ren

Open Case | Open Chart | Print AVS | SnapSh

Filter by Status | Total: 0

ime ▲ | Patient

- Administrative Policy Manual
- Ambulatory Protocols
- Antimicrobial Stewardship
- Bug Book
- Call List
- CDC Website
- Chain of Responsbility
- Chase Paymenttech
- CLABSI Toolkit
- Clinical Care Guidelines
- COG WEBSITE
- Communication Directory
- COVID-19 AllConnect (Lurie Children's intranet)
- COVID-19 Microsoft Sway Resource
- COVID-19 Resources on LurieChildrens.org
- CRU Study Protocols
- DynaMed
- CSF-EncoderPro
- Emergency Response Resources
- Epic Essentials
- Extravasation
- Feinberg Education Portal
- Formulary
- Galter Library
- I-CARE Portal
- IL Prescription Monitoring Program
- Insurance Issue Tracker
- Insurance Prior Authorization
- KidsHealth
- Lab Tests
- Locus Home Monitoring
- Med Abbrev
- Medication Schedule Adjustment Guide
- MedlinePlus
- Micromedex/Poisondex
- NAKI Med List
- NMH Web Paging
- Nursing Policy Manual
- Nutrition
- Pain Management Pocket Guide (UNDER REVISION)

Documents

Prescribing Resources

Category | Name

- + : 01-Optimizing Specimen Collection (2)
- + : 02-Clinical Care Guidelines (16)
- + : 03-Institutional Antibiotic Susceptibilities (1)
- + : 04-Dosing Resources (1)
- + : 05-Interpreting Rapid Diagnostics (3)
- + : 06-True pathogen vs. Contamination/Colonization (1)
- + : 07-Pharmacokinetics & Pharmacodynamics (1)
- + : 08-Therapeutic Drug Monitoring (1)
- + : 09-Antimicrobial Adverse Events (2)
- + : 10-Choosing Duration of Therapy (1)
- + : 11-Perioperative Prophylaxis (5)
- + : 12-Communicating with Families (5)

Antimicrobial Utilization

Category | Name

- + : A01-ASP Documents (2)
- + : A02-Benchmarking (1)
- + : A03-Lurie Trends (37)
- + : A04-Restricted Antimicrobials (2)

Education

Category | Name

- + : E01-Public Health (1)
- + : E02-Resistance Mechanisms (1)
- + : E03-Spectrum of Activity of Antimicrobials (4)
- + : E04-Susceptibilty Test (2)

Links

URL		Notes
**- : Scholarship and Advocacy** (2)		
Areas of Study and Publications	...	
Collaborations	...	
**- : Lurie Children's Hospital Days of Therapy by Department** (1)		
Lurie Children's Hospital Days of Therapy (DOT) by Department	...	
**- : Additional Resources** (11)		
Antimicrobial Susceptibility Report	...	Antibiogram
ASP Article of the Month	...	
ASP Handbook-2021	...	
CDC Core Elements	...	
Health Care Without Harm	...	Public Health
IDSA	...	
Illinois Department of Public Health	...	Public Health
Infection Control Manual	...	
Joint Commission Guidelines	...	
U.S.PIRG	...	Public Health
World Health Organization	...	Public Health

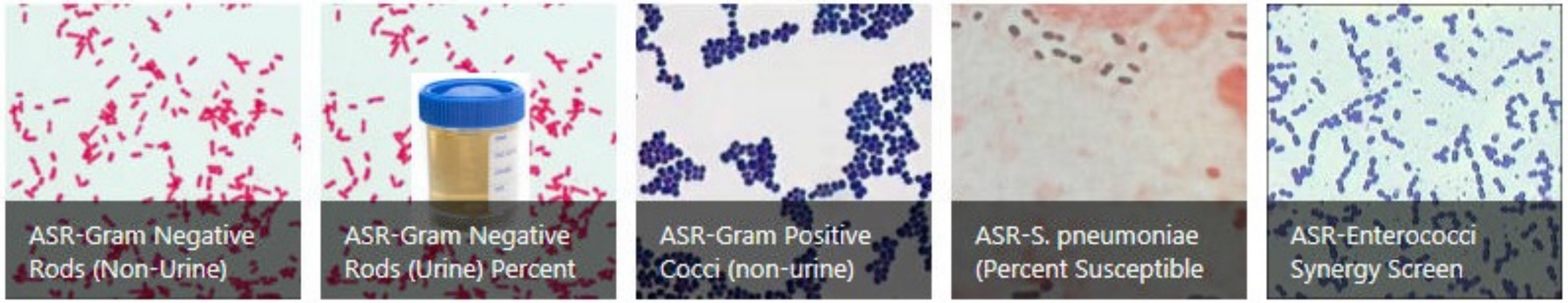

Antibiogram

- Collection of yearly culture and susceptibility results available on Epic
- Used to determine the narrowest, most reasonable empiric therapy

Gram Positive Cocci (non-urine)	No. of Isolates	Penicillin	Ampicillin	Ampicillin/Sulbactam	Amox / Clav	Cefazolin	Cefepime	Ceftriaxone	Cefotaxime	Clindamycin	Azithromycin	Erythromycin	Gentamicin	Levofloxacin	Oxacillin	Rifampin	Linezoli
Streptococcus pneumoniae	37	100/79*						100/97*	100/94*	94	61			97			
Streptococcus mitis/oralis	18	67					78	89	89	94				88			
Other Streptococcus spp. viridans group	28	79					96	96	95	82				95			
Streptococcus pyogenes (Grp A)	53	100					100	100		94		92					
Streptococcus agalactiae (Grp B)	8	100	100							57		38					
*Enterococcus faecalis**	81		99											95		57	98
*Enterococcus faecium**	13		83											45		33	100
Staphylococcus aureus - MSSA	573	25		100	100	100				71		60	98	93	100	99	100
Staphylococcus aureus - MRSA	131	0		0	0	0				65		18	94	52	0	96	100
Staphylococcus epidermidis	96	7		27	27	27				42		15	63	67	28	97	100
Other *Staphylococcus* species	39	21		46	46	46				54		34	91	91	44	97	100

Ann & Robert H. Lurie Children's Hospital of Chicago®

Antibiogram

- Cephalexin = Covers 100% of MSSA (573), 0% MRSA (0) → 81% of all
- Clindamycin = Covers 71% of MSSA (407), 65% MRSA (85) → 70% of all

Gram Positive Cocci (non-urine)	No. of Isolates	Penicillin	Ampicillin	Ampicillin/Sulbactam	Amox / Clav	Cefazolin	Cefepime	Ceftriaxone	Cefotaxime	Clindamycin	Azithromycin	Erythromycin	Gentamicin	Levofloxacin	Oxacillin	Rifampin	Linezoli
Streptococcus pneumoniae	37	100/79*						100/97*	100/94*	94	61			97			
Streptococcus mitis/oralis	18	67					78	89	89	94				88			
Other Streptococcus spp. viridans group	28	79					96	96	95	82				95			
Streptococcus pyogenes (Grp A)	53	100					100	100		94		92					
Streptococcus agalactiae (Grp B)	8	100	100							57		38					
*Enterococcus faecalis**	81		99											95		57	98
*Enterococcus faecium**	13		83											45		33	100
Staphylococcus aureus - MSSA	573	25		100	100	100				71		60	98	93	100	99	100
Staphylococcus aureus - MRSA	131	0		0	0	0				65		18	94	52	0	96	100
Staphylococcus epidermidis	96	7		27	27	27				42		15	63	67	28	97	100
Other *Staphylococcus* species	39	21		46	46	46				54		34	91	91	44	97	100

Northwestern Medicine®
Feinberg School of Medicine

Cefazolin/Ancef (IV), Cephalexin/Keflex (PO)

81% coverage of *S. aureus*

Clindamycin (IV/PO)

70% coverage of *S. aureus*

- Spectrum: Gram positive
- Spectrum: Gram positive cocci

Skin/Soft Tissue Infection			
Impetigo	S. aureus, GAS	Mupirocin topical BID<br><br><u>If multiple lesions or in outbreaks to reduce transmission</u><br>Cephalexin 50 mg/kg/day PO divided TID	Duration 5-7 days
Erysipleas	GAS and other beta-hemolytic Streptococci	Cephalexin 16.7 mg/kg/dose PO TID<br><br><u>Severe B-lactam allergy</u><br>Clindamycin 10 mg/kg/dose every 8 hours (max dose 600 mg) OR	Duration 5-7 days
Cellulitis *w/o purulence*	GAS and other beta-hemolytic Streptococci,	<u>Outpatient:</u> Cephalexin 16.7 mg/kg/dose PO TID<br><br><u>Inpatient:</u> Cefazolin 25 mg/kg/day IV q8h	Duration 5-7 days

- Skin/Soft Tissue Infection
- Simple Cystitis, Pyelonephritis
- Poor oral bioavailability
- High oral bioavailability
- Significant increased risk of *Clostridium difficile*

Minimum Inhibitory concentration

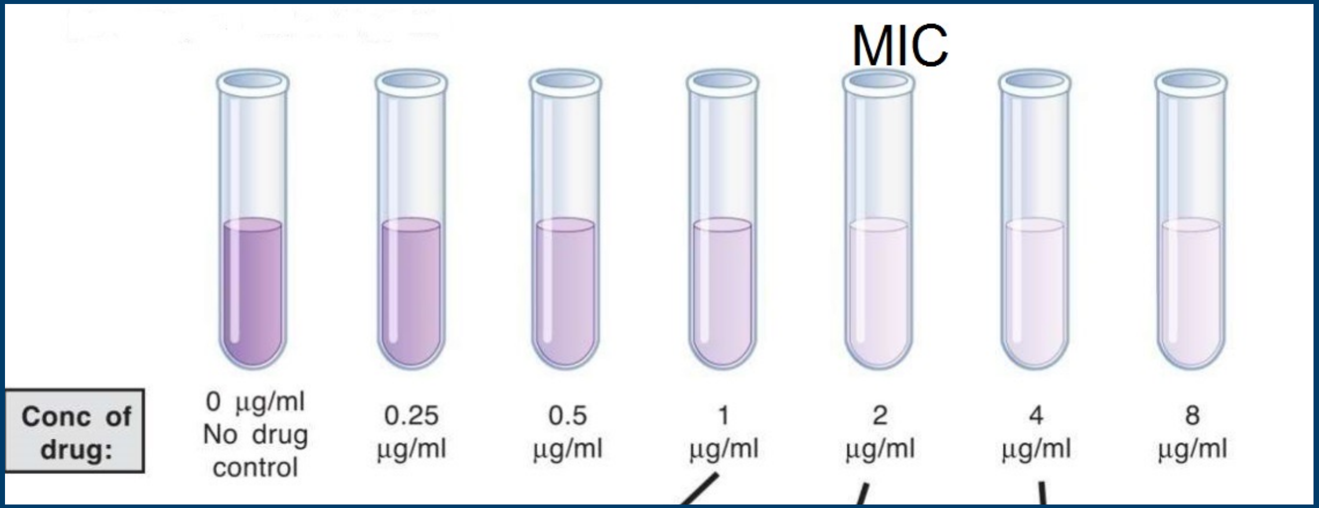

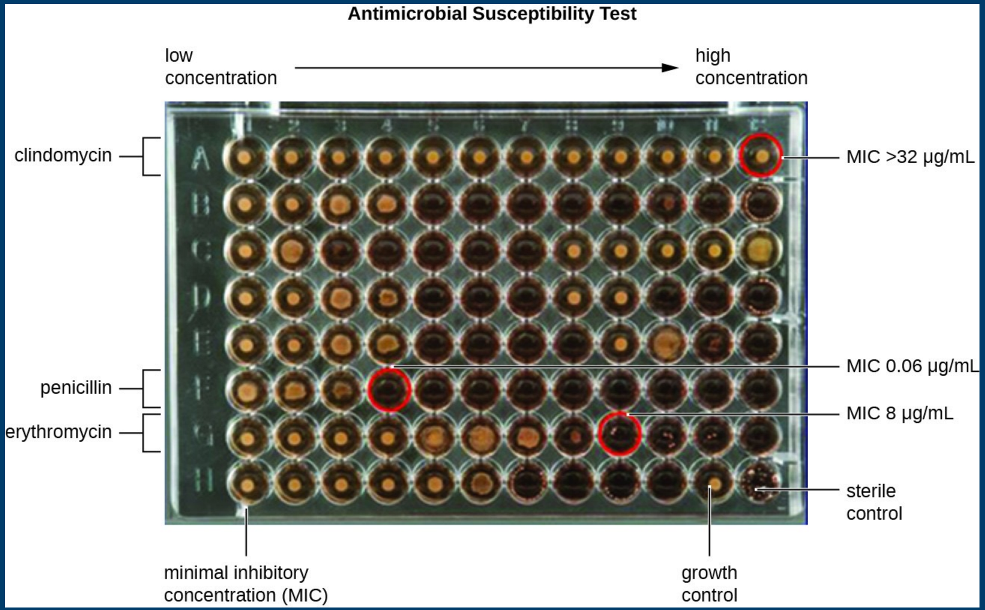

Antimicrobial Agent	*Staphylococcus* spp. Indications	Disk Content	Zone Diameter Breakpoints, nearest whole mm				MIC Breakpoints, µg/mL			
			S	SDD	I	R	S	SDD	I	R
PTIDES										
ests should be performed to determine the susceptibility of all isolates of staphylococci to vancomycin. The disk test does *S. aureus* from vancomycin-intermediate isolates, nor does the test differentiate among vancomycin-susceptible, -intermediate, han *S. aureus*, all of which give similar size zones of inhibition.										
Vancomycin	*S. aureus*	–	–	–	–	–	≤2	–	4–8	≥16

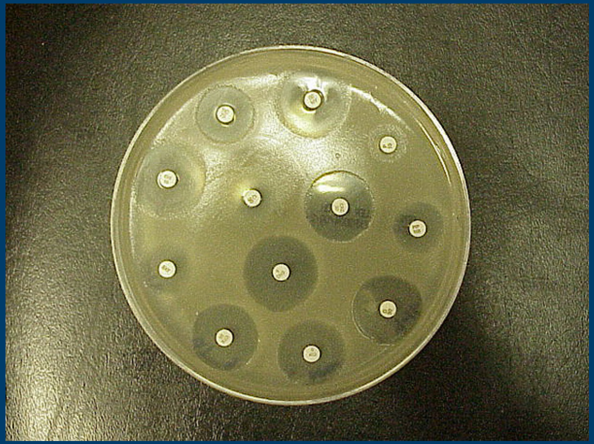

TMP-SMX/Bactrim (IV/PO)

- Spectrum: Gram positive cocci, gram negative rods
  - MRSA/MSSA, Group A *Strep*
- Common indications
  - Skin/Soft Tissue Infection
  - Simple Cystitis, Pyelonephritis
  - PJP Prophylaxis/Disease

Antibiotic	MIC	Interpretation
**Amoxicillin/Clavulanate**	16/4	Resistant
**Ampicillin/Sulbactam**	32/16	Resistant
**Cefazolin**	8	Resistant
**Clindamycin**	8	Resistant
**Gentamicin**	≤ 1	Susceptible
**Levofloxacin**	≤ 0.5	Susceptible
**Vancomycin**	1	Susceptible
**Meropenem**	8	Resistant
**Oxacillin**	8	Resistant
**Penicillin**	8	Resistant
**TMP-SMX**	≤ 0.5/9.5	Susceptible
**Daptomycin**	≤ 0.5	Susceptible
**Linezolid**	2	Susceptible
**Tetracycline**	≤ 1	Susceptible
