# Supplementary material for: Antimicrobial stewardship for nurse practitioners and physician assistants: enhancing patient safety through education
Source: Antimicrob Steward Healthc Epidemiol. 2023 Oct 6;3(1):e165. doi: 10.1017/ash.2023.434 (PMC10644159; doi:10.1017/ash.2023.434)
Supplement: Parzen-Johnson et al. supplementary material 2 — Parzen-Johnson et al. supplementary material [file S2732494X23004345sup002.pdf]

Problem-Based Learning Exercise:

A 9yo male comes in the emergency room with a new rash. He is well appearing and denies any recent fever. His left thigh has a 1cm area of fluctuance and a 3cm area of surrounding erythema. The site has warmth, tenderness to palpation and mild edema. He has no other physical exam findings currently. You make a diagnosis of community acquired purulent cellulitis. You perform an incision and drainage and send the sample for culture. What is the most common organism to cause this infection?

A. *Escherichia coli*
B. *Proteus mirabilis*
C. *Pseudomonas aeruginosa*
D. *Staphylococcus aureus*
E. *Staphylococcus epidermidis*

Gram Positive Cocci (non-urine)	No. of Isolates	Penicillin	Ampicillin	Ampicillin/Sulbactam	Amox / Clav	Cefazolin	Cefepime	Ceftriaxone	Cefotaxime	Clindamycin	Azithromycin	Erythromycin	Gentamicin	Levofloxacin	Oxacillin	Rifampin	Linezoli
Streptococcus pneumoniae	37	100/79*						100/97*	100/94*	94	61			97			
Streptococcus mitis/oralis	18	67					78	89	89	94				88			
Other Streptococcus spp. viridans group	28	79					96	96	95	82				95			
Streptococcus pyogenes (Grp A)	53	100					100	100		94		92					
Streptococcus agalactiae (Grp B)	8	100	100							57		38					
*Enterococcus faecalis**	81		99											95		57	98
*Enterococcus faecium**	13		83											45		33	100
Staphylococcus aureus - MSSA	573	25		100	100	100				71		60	98	93	100	99	100
Staphylococcus aureus - MRSA	131	0		0	0	0				65		18	94	52	0	96	100
Staphylococcus epidermidis	96	7		27	27	27				42		15	63	67	28	97	100
Other *Staphylococcus* species	39	21		46	46	46				54		34	91	91	44	97	100

Based on the Lurie institutional antibiogram, approximately what percentage of *Staphylococcus aureus* isolates are resistant to methicillin or 1st generation cephalosporins (i.e. Keflex/cephalexin, Ancef/cefazolin)?

A. 10%
B. 20%
C. 50%
D. 80%
E. 100%

What percentage of *Staphylococcus epidermidis* are susceptible to levofloxacin?

A. 52%
B. 67%
C. 91%
D. 93%
E. 100%

You decide that you will discharge this patient on oral antibiotics for a 5-day course of therapy. What is the most appropriate antibiotic for this patient?

A. Bactrim/TMP-SMX
B. Clindamycin
C. Keflex/Cephalexin
D. Linezolid
E. Vancomycin

Two days later, you receive a phone call from the microbiology lab informing you that your culture is positive for *Staphylococcus aureus*. The report includes the following susceptibility report:

Antibiotic	MIC	Interpretation
Amoxicillin/Clavulanate	$\leq 4/2$	Susceptible
Ampicillin/Sulbactam	$\leq 8/4$	Susceptible
Cefazolin	≤ 4	Susceptible
Clindamycin	8	Resistant
Gentamicin	≤ 1	Susceptible
Levofloxacin	≤ 0.5	Susceptible
Vancomycin	1	Susceptible
Meropenem	≤ 2	Susceptible
Oxacillin	≤ 0.25	Susceptible
Penicillin	8	Resistant
TMP-SMX	$\leq 0.5/9.5$	Susceptible
Daptomycin	0.5	Susceptible
Linezolid	2	Susceptible
Tetracycline	≤ 1	Susceptible

Which of the following statements is true?

A. Daptomycin (MIC 0.5) is superior to vancomycin (MIC 1) for this infection because of a lower MIC
B. This bacterium can be characterized as methicillin resistant *Staphylococcus aureus* (MRSA)
C. Your original choice of antibiotic for this patient needs to be altered for appropriate therapy
D. Cephalexin is an appropriate antibiotic for this bacterial isolate
E. Levofloxacin is a better choice for this infection because it has a narrower spectrum of activity than your original choice of antibiotic
F. A & D
G. A & C
