# Supplementary material for: Antimicrobial stewardship for nurse practitioners and physician assistants: enhancing patient safety through education
Source: Antimicrob Steward Healthc Epidemiol. 2023 Oct 6;3(1):e165. doi: 10.1017/ash.2023.434 (PMC10644159; doi:10.1017/ash.2023.434)
Supplement: Parzen-Johnson et al. supplementary material 3 — Parzen-Johnson et al. supplementary material [file S2732494X23004345sup003.pdf]

Pre-Assessment

The following survey is meant to assess both your comfort and knowledge related to antimicrobial stewardship. These answers are not part of any Lurie Children's Hospital formal review process and will be used for research purposes only to assess efficacy of the Antimicrobial Stewardship APP Teaching curriculum:

Thank you for your participation!

Simon Parzen-Johnson, MD - APN Education Series - IRB 2022-5033
Completing the survey is voluntary and any question may be skipped
The purpose of this study is to assess methods of teaching antimicrobial stewardship skills to APP's at Lurie Children's Hospital
Any anonymous information from the survey, once entered into the database cannot be removed
There are no perceived risks in participating, however given that email will be collected on the Redcap database, if that database was compromised in some way, this information could be disseminated
By filling out the survey, consent is implied to be a participant in the study
Contact Information: sparzenjohnson@luriechildrens.org

Upon completion of this survey, a second assessment will be sent out to you in one week and in six months to the email included. Although you will receive emails to complete follow up surveys, these emails will not be reviewed and individual responses will never be reviewed separately. All results will be assessed in aggregate.

---

1) Email: ______________________________

---

2) What is your current job title in the hospital?

- ◯ APN
- ◯ PA
- ◯ RN
- ◯ Other

---

3) How many years (to the closest year) have you worked in your current role? ______________________________

4) What division within the hospital system are you a member of?

- ◯ Adolescent & Young Adult Medicine
- ◯ Advanced General Pediatrics &Primary Care
- ◯ Allergy & Immunology
- ◯ Anesthesiology
- ◯ Brain Tumor Program
- ◯ Cancer & Blood Disorders
- ◯ Cardiology & Cardiac Surgery
- ◯ Cleft Lip & Palate Repair Program
- ◯ Critical Care
- ◯ Dermatology
- ◯ Developmental & Behavioral Pediatrics
- ◯ Emergency Medicine
- ◯ Endocrinology
- ◯ ENT
- ◯ Epilepsy Program
- ◯ Gastroenterology & GI Surgery
- ◯ Gender & Sex Development Program
- ◯ Birth Defects & Metabolism
- ◯ Hospital-Based Medicine
- ◯ Infectious Diseases
- ◯ Medical Imaging
- ◯ Neonatology
- ◯ Nephrology
- ◯ Neurology
- ◯ Neurosurgery
- ◯ Ophthalmology
- ◯ Orthopaedics
- ◯ Palliative Care
- ◯ Plastic & Reconstructive Surgery
- ◯ Primary Care Pediatrics
- ◯ Psychiatry
- ◯ Pulmonary Medicine
- ◯ Rheumatology
- ◯ Spina Bifida Center
- ◯ Sports Medicine
- ◯ Surgery
- ◯ Transplant Surgery
- ◯ Urology
- ◯ Wellness & Weight Management Program

For the following statements, indicate how effective your institution is in teaching you:

	Not at all effective	Somewhat effective	Moderately effective	Very effective	Extremely effective	N/A
5) The importance of antimicrobial stewardship in the inpatient setting	◯	◯	◯	◯	◯	◯
6) The importance of antimicrobial stewardship in the outpatient setting	◯	◯	◯	◯	◯	◯
7) The importance of educating patients on antimicrobial stewardship	◯	◯	◯	◯	◯	◯

For the following statements, indicate how confident you are in your ability to:

	Not at all confident	Somewhat confident	Moderately confident	Very confident	Extremely confident	N/A
8) Practice antimicrobial stewardship in the inpatient setting	◯	◯	◯	◯	◯	◯
9) Practice antimicrobial stewardship in the outpatient setting	◯	◯	◯	◯	◯	◯
10) Choose the right empiric antibiotics prior to culture results	◯	◯	◯	◯	◯	◯
11) Interpret antibiotic susceptibility results for a bacterial culture	◯	◯	◯	◯	◯	◯

The following questions are related to knowledge about antimicrobial stewardship and appropriate antibiotic use:

12) What is an antibiogram?

◯ a. A standard 1g dose of antibiotics
◯ b. A collection of susceptibility results for bacteria
◯ c. A collection of appropriate dosing for antibiotics
◯ d. A collection of which bacteria cause common infections
◯ e. A collection of which antibiotics to use for common infections

---

13) The minimum inhibitory concentration (MIC) helps determine what?

◯ a. The highest concentration of antibiotic that inhibits growth of bacteria
◯ b. The lowest concentration of antibiotic that inhibits growth of bacteria
◯ c. The lowest concentration of antibiotic that kills bacteria
◯ d. The highest concentration of antiviral that inhibits growth of viruses

---

14) What are the two most common bacteria that cause cellulitis?

◯ a. Staphylococcus aureus and Group A Streptococcus
◯ b. Staphylococcus aureus and Streptococcus pneumoniae
◯ c. Staphylococcus epidermidis and Group A Streptococcus
◯ d. Staphylococcus epidermidis and Streptococcus pneumoniae
◯ e. Staphylococcus aureus and Staphylococcus epidermidis

---

15) According to resistance patterns from Lurie Children's Hospital, which of these antibiotics covers a higher percentage of Staphylococcus aureus bacteria isolates

◯ a. Cephalexin
◯ b. Clindamycin

---

16) What is the most appropriate first-line antibiotics for simple community-acquired cellulitis?

◯ a. Clindamycin
◯ b. Cephalexin (Keflex)
◯ c. Linezolid
◯ d. TMP-SMX (Bactrim)
◯ e. Ceftriaxone

17) Which of the following statments about the list of antibiotic susceptibilities for an isolate of Staphylococcus aureus is true?

Antibiotic MIC Interpretation
Amoxicillin/Clavulanate ≤4/2 Susceptible
Ampicillin/Sulbactam ≤8/4 Susceptible
Cefazolin ≤4 Susceptible
Clindamycin ≤0.25 Susceptible
Gentamicin ≤1 Susceptible
Levofloxacin ≤0.5 Susceptible
Vancomycin 1 Susceptible
Meropenem ≤2 Susceptible
Oxacillin ≤0.25 Susceptible
Penicillin 8 Resistant
TMP-SMX ≤0.5/9.5 Susceptible
Daptomycin 0.5 Susceptible
Linezolid 2 Susceptible
Tetracycline ≤1 Susceptible

◯ a. Linezolid (MIC 2) is inferior to vancomycin (MIC 1) for this infection because of a higher MIC
◯ b. This bacterium can be characterized as methicillin resistant Staphylococcus aureus (MRSA)
◯ c. Daptomycin (MIC 0.5) is superior to vancomycin (MIC 1) for this infection because of a lower MIC
◯ d. Clindamycin and cefazolin would both be effective in treating this infection
◯ e. Levofloxacin is a better choice for this infection because it has the narrowest spectrum of activity
