# Supplementary material for: Antimicrobial stewardship for nurse practitioners and physician assistants: enhancing patient safety through education
Source: Antimicrob Steward Healthc Epidemiol. 2023 Oct 6;3(1):e165. doi: 10.1017/ash.2023.434 (PMC10644159; doi:10.1017/ash.2023.434)
Supplement: Parzen-Johnson et al. supplementary material 4 — Parzen-Johnson et al. supplementary material [file S2732494X23004345sup004.pdf]

S4 Breakdown of Specialties for Participants	
**Specialty**	**Number**
Advanced General Pediatrics & Primary Care	2
Allergy & Immunology	1
Anesthesiology	2
Brain Tumor Program	1
Cancer & Blood Disorders	8
Cardiology & Cardiac Surgery	22
Critical Care	6
Emergency Medicine	5
Endocrinology	1
ENT	1
Gastroenterology & GI Surgery	2
Hospital-Based Medicine	2
Infectious Diseases	2
Neonatology	5
Nephrology	2
Neurology	1
Orthopedics	1
Palliative Care	1
Plastic & Reconstructive Surgery	1
Primary Care Pediatrics	1
Pulmonary Medicine	2
Surgery	8
Transplant Surgery	1
Urology	1
Left Blank	1
